# Long-Term Survival and Late Toxicity Associated With Pelvic Intensity Modulated Radiation Therapy (IMRT) for Cervical Cancer Involving CT-Based Positive Lymph Nodes

**DOI:** 10.3389/fonc.2019.00520

**Published:** 2019-06-19

**Authors:** Chengzhi Lei, Shaokang Ma, Manni Huang, Jusheng An, Bin Liang, Jianrong Dai, Lingying Wu

**Affiliations:** ^1^Department of Gynecologic Oncology, National Cancer Center, National Clinical Research Center for Cancer, Cancer Hospital, Chinese Academy of Medical Sciences, Peking Union Medical College, Beijing, China; ^2^Department of Radiation Oncology, National Cancer Center, National Clinical Research Center for Cancer, Cancer Hospital, Chinese Academy of Medical Sciences, Peking Union Medical College, Beijing, China

**Keywords:** cervical cancer, intensity modulated radiation therapy, intracavitary brachytherapy, long-term survival, late toxicity

## Abstract

The purpose of this study was to evaluate the outcomes and toxicity experienced by cervical cancer patients with positive lymph nodes (LNs) who were treated with intensity-modulated radiation therapy (IMRT) and intracavitary brachytherapy (ICBT) plus concurrent chemotherapy. We retrospectively evaluated 108 cervical cancer patients with computed tomography (CT)-based positive LNs treated with IMRT and ICBT plus concurrent chemotherapy between 2009 and 2011. IMRT plans were designed to deliver 50 Gy to 95% of the planning target volume (PTV; cervical tumor, pelvis, and parametrium), with daily doses of 1.6–1.8 and 60–70 Gy to 95% of the planning gross tumor volume (PGTV)-LN (pelvic or para-aortic LNs), with daily doses of 2.0–2.2 Gy. Overall survival (OS) and progression-free survival (PFS) Kaplan–Meier curves were plotted. Acute and late toxicities were evaluated according to the Radiation Therapy Oncology Group and European Organization for Research and Treatment of Cancer toxicity criteria. Of the 108 cases, 45 were stage IIB and 63 were stage IIIB. The median follow-up was 65 months (range 2–83). Overall, the 5 year cumulative incidences of pelvic failure alone, distant failure alone, and synchronous pelvic and distant failure were 8.3, 12.9, and 8.3%, respectively. The 5 year OS rate was 67.6%, and the 5 year PFS rate was 53.7%. The 5 year cumulative incidence was 9.2% for late gastrointestinal and genitourinary toxicities of Grade ≥3 and 51.8% for acute leukopenia of Grade ≥3. The clinical results suggest that IMRT and ICBT with concurrent chemotherapy is an effective treatment, with acceptable toxicity, for advanced cervical cancer involving positive LNs.

## Introduction

Over the past decades, numerous studies have shown that intensity-modulated radiation therapy (IMRT) and intracavitary brachytherapy (ICBT) with concurrent chemotherapy is a treatment option for patients with locally advanced cervical cancer (1, 2). Compared with conventional external beam radiation therapy (EBRT; using a 3–4-field box technique), the higher target conformity of IMRT helps to deliver a sufficient dose to locoregional lymph nodes (LNs) while limiting the dose delivered to pelvic and abdominal organs at risk (OARs), including the bowel, rectum, bladder, and bone marrow, thereby lowering the incidences of early and late gastrointestinal (GI), genitourinary (GU), and hematologic toxicity (3, 4).

Despite the promising results of IMRT for cervical cancer, outcome data remains limited. Moreover, the effects of IMRT and ICBT with concurrent chemotherapy in women with cervical cancer and an intact uterus, particularly for stage IIB and IIIB patients with positive LNs, remain unclear. Studies reporting long-term outcomes and toxicities are needed.

## Materials and Methods

### Patients

Between November 2009 and November 2011, 116 stage IIB and IIIB cervical cancer patients with computed tomography (CT)-based positive LNs were treated with IMRT and ICBT plus concurrent chemotherapy at our institution. Eight patients were lost to follow-up by November 2016. The clinical data (up to November 2016) of the remaining 108 patients were retrospectively analyzed in this study.

The pre-treatment workup included medical history, gynecologic pelvic examination, chest radiography, or CT scan, complete blood count, and blood chemistry profile, including squamous cell carcinoma (SCC) antigen level. All patients had histologically confirmed cervical tumors. Clinical stage was determined based on consensus between the gynecologic oncologists. Abdominopelvic CT or magnetic resonance imaging (MRI) images were used for workup and treatment planning. The patients' clinicopathologic characteristics are shown in Table 1.

**Table 1:** Patient characteristics.

**Characteristic**		***N* = 108(%)**
Age (years)		
Mean	49	
Range	31–73	
SCC antigen level (median)	16.8 ng/ml	
FIGO stage		
IIB		45 (41.7)
IIIB		63 (58.3)
Histology		
SCC		105 (97.2)
Adenocarcinoma		2 (1.9)
Adenosquamous carcinoma		1 (0.9)
Tumor grade		
Well-differentiated		2 (1.9)
Moderately differentiated		44 (40.7)
Poorly differentiated		20 (18.5)
Moderate to poorly differentiated		7 (6.5)
differentiated		
Unknown		35 (32.4)
Nodal involvement		
Pelvic nodes		74 (68.5)
Para-aortic nodes		4 (3.7)
Pelvic + para-aortic nodes		18 (16.7)
Inguinal + pelvic nodes		5 (4.6)
Others		7 (6.5)

*FIGO, International Federation of Gynecology and Obstetrics; SCC, squamous cell carcinoma*.

### IMRT Techniques

All patients underwent IMRT based on full-bladder CT-based planning with custom immobilization, intravenous contrast media, and a slice thickness of 5 mm. The clinical target volume (CTV) comprised the cervix, parametrium, uterus, upper third to a half of the vagina, and regional LNs (internal, external iliac, and common). In women with involvement of the lower third of the vagina, inguinal nodes were also treated (5, 6). In patients without para-aortic LN metastases, the upper field border was at the level of the L4/L5 interspace. In patients with para-aortic LN metastases, the upper border was up to the level of the renal vessels or three slices (15 mm) above the macroscopic LN metastases. The caudal field border was at the lower margin of the obturator foramen or at the lowest extension of the tumor (inferiorly). The gross tumor volume (GTV) comprised the cervical tumor, enlarged LNs, and metastases in any region. Accounting for organ motion and setup uncertainty, we applied a 0–3 mm margin around the CTV to create the planning target volume (PTV), and a 0–5 mm margin around the GTV to create the planning gross tumor volume (PGTV).

The IMRT plans consisted of 3–7 coplanar fields with 6 MV photon beams. The prescription doses to cover 95% of the PTV and PGTV were 45–50 and 60–70 Gy, respectively. The maximum doses delivered to the PTV and PGTV were <110% of the prescription doses. Each IMRT plan involved 28–30 fractions (over 5 weeks). The daily doses delivered to the PTV and PGTV were 1.5–1.8 and 2.0–2.4 Gy, respectively.

The following OARs were delineated: spinal cord, femoral heads, kidneys, bladder, rectum, small bowel, and pelvic bone marrow. The delineation of the small bowel exceeded the upper and lower border of the PTV by two slices. The OAR planning constraints were as follows: (1) rectum: maximal dose <60 Gy, volume receiving >50 Gy (V_50_) <20%; (2) bowel: maximal dose <52 Gy, V_40_ <60%; (3) bladder: V_50_ <20%; and (4) intestines: maximal dose <52 Gy, V_40_ <50%.

Patients who were undergoing definitive IMRT received Ir^192^ high-dose-rate ICBT insertions, with a total dose of 35–42 Gy (5–6 fractions weekly, at 7 Gy each) delivered to Point A. For patients with a bulky tumor or a tumor involving the upper third of the vagina, a total dose of 10–22 Gy (1–2 fractions) to 0.5 cm beneath the vaginal mucosa was delivered using a vaginal ovoid applicator before beginning the ICBT involving a tandem applicator. An overview of the radiotherapy characteristics is presented in Table 2.

**Table 2:** Treatment characteristics.

**Characteristic**	**Median dose**	***N* = 108 (%)**
IMRT		
PTV	50.4 ± 1.8 Gy	
PGTV	61.6 ± 3.3 Gy	
ICBT delivered to point A	35 ± 2.8 Gy	
Chemotherapy regimens		
Cisplatin		49 (45.4)
Paclitaxel + cisplatin		44 (40.7)
Paclitaxel + carboplatin		7 (6.5)
Cisplatin + 5-fluorouracil		2 (1.9)
Others		6 (5.5)

### Chemotherapy

Just under half of the patients were treated with concurrent weekly cisplatin monotherapy (35–40 mg/m^2^). Alternative regimens included weekly cisplatin plus 5-fluorouracil; weekly paclitaxel (45–50 mg/m^2^) plus cisplatin (40 mg/m^2^); paclitaxel (175 mg/m^2^) plus cisplatin (50 mg/m^2^) every 3 weeks; or paclitaxel (175 mg/m^2^) plus carboplatin at a dose leading to an area under the concentration- vs. time- curve (AUC) of two, every 3 weeks. The patients' chemotherapy regimens are shown in Table 2.

### Toxicities

Acute and late toxicities were evaluated according to the Radiation Therapy Oncology Group (RTOG) and European Organization for Research and Treatment of Cancer (EORTC) toxicity criteria (7, 8). A toxicity was classed as acute if it occurred during treatment or within the first 3 months after treatment, and late if it occurred after 3 months. Acute toxicities were evaluated weekly during treatment, and at 6 weeks and 3 months after treatment. Late toxicities were evaluated 6 months after treatment and once a year thereafter. Evaluations of toxicities were performed by an experienced radiation oncologist.

### Follow-Up

Follow-up evaluation included physical examination, SCC antigen level, blood counts, and B scan abdominopelvic CT and/or positron emission tomography (PET)-CT scans if necessary. The initial tumor response was evaluated by an experienced gynecologic oncologist at 3 months after treatment and every 3 months thereafter. Outcome events were measured from the time after treatment.

Overall survival (OS) was defined as the time to death from any cause. Pelvic failure and distant failure were defined as the time to the first radiographic and/or pathologic evidence of disease recurrence in or outside the pelvis, respectively. Progression-free survival (PFS) was defined as the time to the first evidence of disease recurrence or death from any cause. Patients without a PFS event were censored at the last known medical encounter. Time to late toxicity was measured from the completion of radiotherapy. Survival curves were plotted using the Kaplan–Meier method. All analyses were carried out using SPSS version 19.0.

## Results

### Patients

Of the 108 patients, 45 (41.7%) had stage IIB cancer and 63 (58.3%) had stage IIIB cancer; 105 patients had SCC, two had adenocarcinoma, and one had adenosquamous carcinoma. The median doses delivered to the PTV and PGTV were 50.4 and 61.6 Gy, respectively. The median dose delivered to Point A was 35 Gy. Concurrent chemotherapy was prescribed for all patients: 49 (45.4%) patients received cisplatin monotherapy, 44 (40.7%) received paclitaxel/cisplatin, seven received paclitaxel/carboplatin, two (1.9%) received cisplatin/5-fluorouracil, and six received other regimens.

### Outcomes and Locoregional Control

**Figure 1:**
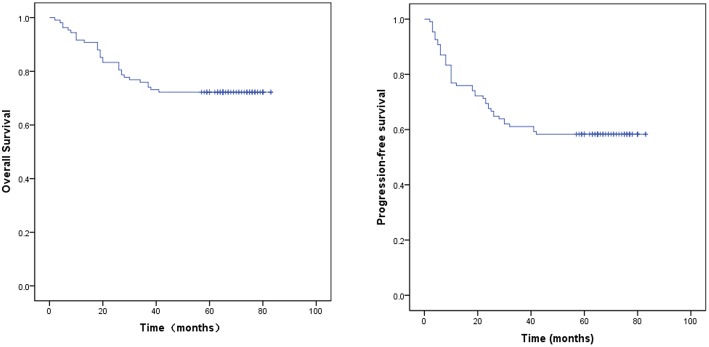
Kaplan–Meier curves of progression-free survival (PFS) and overall survival (OS).

The median follow-up time for the surviving patients was 65 months. For all patients, the 1, 3, and 5 year OS rates were 91.7, 75.9, and 67.6%, respectively. The 1, 3, and 5 year PFS rates were 76.9, 61.1, and 53.7%, respectively. For stage IIB patients, the 5 year OS and PFS rates were 73.3 and 68.9%, respectively. For stage IIIB patients, the 5 year OS and PFS rates were 63.4 and 42.9%, respectively. The OS and PFS curves are shown in Figure 1. Cox proportional hazard model analysis showed that tumor size and age were independent prognostic factors for OS (*P* < 0.05) (Table 3).

**Table 3:** Cox proportional hazard analysis of variables predicting overall survival (OS).

	**P**	**RR**	**95% CI**
Age	0.003	1.067	1.022–1.113
Stage	0.578	1.280	0.536–3.058
Cervical tumor size	0.032	1.445	1.033–2.021
Nodal tumor size	0.765	1.028	0.855–1.237
Node tumor location	0.963	1.007	0.747–1.357
SCC level	0.230	1.0112	0.992–1.033

### Patterns of Failure

The 5 year cumulative incidences of pelvic failure alone, distant failure alone, and synchronous pelvic and distant failure were 8.3% (9/108), 12.9% (14/108), and 8.3% (9/108), respectively. Pelvic failure locations included the cervix in patients, vagina in patients, pelvic LNs in patients, and cervix plus LNs in patients. The 5 year cumulative incidences of distant failure alone for stage IIB and IIIB patients were 4.4% (2/45) and 19% (12/63), respectively.

### Toxicity

The 5 year cumulative incidence of late toxicity of Grade ≥2 was 42.5%. The median time to late toxicity was 10–16 months. Late Grade 3 GI toxicity occurred in 8.3% of patients with rectovaginal fistulas in nine patients. Late Grade 2 GI toxicity occurred in 27 patients with small bowel obstructions. Late Grade 3 GU toxicity occurred in 0.9% of patients with vesicovaginal fistulas in one patient. Late Grade 2 GU toxicity occurred in nine patients with hematuria requiring cystoscopy in all patients with late toxicity of Grade ≥ 3 were treated with ICBT. The 5 year cumulative incidence of acute leukopenia of Grade ≥ 3 was 51.8% (Tables 4, 5).

**Table 4:** Incidence of treatment-related acute hematologic toxicity (%).

**Toxicity**	**Grade 1**	**Grade 2**	**Grade 3**	**Grade 4**
Leukopenia	8.3	39.8	48.2	3.7
Thrombocytopenia	55.6	26.9	10.2	1.9
Anemia	53.7	32.4	13.0	0.9

**Table 5:** Incidence of treatment-related late toxicity (%).

**Toxicity**	**Grade 0**	**Grade 1**	**Grade 2**	**Grade 3**
Gastrointestinal	29.6	37	25	8.3
Genitourinary	63.9	26.9	8.3	0.9

## Discussion

Intensity-modulated radiation therapy and ICBT with concomitant chemotherapy (such as cisplatin-based chemotherapy) is a therapy option for locally advanced cervical cancer. LN metastasis is the most important prognostic factor in cervical cancer (9). It has been shown that a high proportion of cervical cancer patients with disease relapse have LN metastases. These unsatisfactory results may be attributable to insufficient doses delivered to the nodal region (especially in cases involving clinically suspected LN metastases), a geographical target miss, or a combination of both factors (10). For advanced cervical cancer, a conventional EBRT boost (involving a 3 to 4-field-box technique), delivered to the gross tumor, has been widely used. However, due to the high level of toxicity, this treatment tends to fail to deliver the necessary dose to treat locoregional LN metastases. In cervical cancer treated with radiation therapy, late GI, and/or GU toxicity remains a clinical concern and a dose-limiting factor. Late high-grade GI toxicity is reported in up to 35% of cervical cancer patients undergoing chemoradiation. Several studies have been published on the role of IMRT for reducing the incidence and severity of GI toxicity in patients with gynecological malignancies.

Examples of pelvic irradiation toxicity include malabsorption, cystitis, myelosuppression, fistulae, strictures, bowel obstruction, and pelvic fractures. The toxicities associated with conventional EBRT involving a 4-field box (4FB) technique in patients with locoregionally advanced cancer are unsatisfactory. Several retrospective studies have demonstrated the advantage of IMRT over the conventional 4FB technique. Mundt et al. found that the rate of late GI toxicity of Grade ≥ 2 was significantly lower for IMRT than for 4FB (3 vs. 20%) (7). For patients treated with X plus concurrent chemotherapy compared to Y, Brixey et al. found that there was a lower rate of acute hematologic toxicity of Grade ≥2 (31 vs. 60%) (11). Hasselle et al. reported that, for patients with an intact cervix, the rates of late GI, GU, and total toxicity of Grade ≥3 were 4.5, 5.6, and 10.1%, respectively (12). Du et al. reported that the rate of leukopenia of Grade 2/3 was 3.5% for IMRT, and the rate of late toxicity of Grade 3/4 was 18.3% for IMRT and 15.0% for 4FB (13).

In our study, for the 108 patients with stage IIB cervical cancer plus enlarged pelvic LNs and IIIB cervical cancer, who were all treated with IMRT and ICBT with concurrent chemotherapy, the 5 year cumulative incidence of late toxicity of Grade ≥2 was 42.5%. Late GI toxicity of Grade 3 (including rectovaginal fistulas and small bowel obstruction) occurred in 8.3% of the patients. Late GU toxicity of Grade 3 (including vesicovaginal fistulas in, hematuria requiring cystoscopy in, and vaginal necrosis in) occurred in 0.9% of the patients. The 5 year cumulative incidence of acute leukopenia of Grade ≥3 was 51.8%. In the RTOG 92-10 study, the rate of acute hematological toxicity was 76% for cervical cancer patients with positive para-aortic LNs, while the rate of late toxicity was 24% (14). Compared to our study, the higher rate of acute hematological toxicity in the RTOG 92-10 study may be attributable to the differences in the chemotherapy regimens, the higher nodal boost doses, and the higher mean point A dose (14).

In our study, all patients with late toxicity of Grade ≥3 were treated with ICBT. Patients received Ir192 high-dose-rate ICBT insertions, with a total dose of 35–42 Gy (5–6 fractions weekly, at 7 Gy each) delivered to Point A. In our hospital IMRT used in cervical cancer treatment is still at groping stage from 2009 to 2011. The brachytherapy dose referenced traditional radiation therapy, which is higher than current ABS guidelines. In recent years, with the improvement of intracavitary radiotherapy technology including prescription to a volume, three-dimensional planning and MRI for target volume delineation, side effects, and toxicity need to be further studied in future.

A systematic review by Veldeman et al. of OS and toxicity showed evidence of reduced toxicity related to IMRT (15). A retrospective study of 109 patients with stage IB2-IVA cervical carcinoma treated with IMRT and concurrent chemotherapy showed that the 3 year OS, local failure-free survival, and disease-free survival rates were 78.2, 78.1, and 67.6%, respectively (16). In another retrospective study of 111 patients with stage I-IVA cervical carcinoma treated with IMRT, the 3 year OS, PFS, pelvic failure, and distant failure rates were 61.4, 51.4, 29.2, and 25%, respectively (17). Du et al. compared IMRT and 4FB for stage IIB and IIIB cervical cancer, and they found no significant difference for the 1 and 3 year OS rates, but significant differences for the 5 year OS rate (71.2 vs. 60.3%) and 5 year PFS rate (64.9 vs. 44.3%) (13).

The data in our study show that the median follow-up time for the surviving patients was 65 months. For all patients, the 1, 3, and 5 year OS rates were 91.7, 75.9, and 67.6%, respectively, and the 1, 3, and 5 year PFS rates were 76.9, 61.1, and 53.7%, respectively. For stage IIB patients, the 5 year OS and PFS rates were 73.3 and 68.9%, respectively. For stage IIIB patients, the 5 year OS and PFS rates were 63.4 and 42.9%, respectively. Cox proportional hazard model analysis showed that tumor size and age were independent prognostic factors for OS. Our results suggest that IMRT and ICBT with concurrent chemotherapy in patients with cervical cancer and pelvic LN metastasis provided favorable treatment outcomes, with acceptable rates of acute and late GI toxicities comparable to those associated with conventional EBRT.

In conclusion, the clinical results from our institute suggest that IMRT and ICBT with concurrent chemotherapy is a feasible treatment for stage IIB and IIIB cervical cancer patients with CT-based positive LNs. Nevertheless, randomized controlled trials are required to further evaluate IMRT and compare it with the conventional EBRT technique.

## Data Availability

No datasets were generated or analyzed for this study.

## Ethics Statement

This study was carried out in accordance with the Declaration of Helsinki and approved by the independent ethics committee of the Cancer Hospital, Chinese Academy of Medical Sciences. The need to obtain informed consent was waived by the independent ethics committee.

## Author Contributions

CL and SM conceived the study and wrote the paper. MH, JA, and BL collected and analyzed the data. JD and LW provided expert clinical knowledge. All authors critically edited the manuscript for important intellectual content.

### Conflict of Interest Statement

The authors declare that the research was conducted in the absence of any commercial or financial relationships that could be construed as a potential conflict of interest.
